# Topology and weights in a protein domain interaction network – a novel way to predict protein interactions

**DOI:** 10.1186/1471-2164-7-122

**Published:** 2006-05-23

**Authors:** Stefan Wuchty

**Affiliations:** 1 Northwestern Institute on Complexity, Northwestern University, 600 Foster Street, Evanston, IL 60208, USA

## Abstract

**Background:**

While the analysis of unweighted biological webs as diverse as genetic, protein and metabolic networks allowed spectacular insights in the inner workings of a cell, biological networks are not only determined by their static grid of links. In fact, we expect that the heterogeneity in the utilization of connections has a major impact on the organization of cellular activities as well.

**Results:**

We consider a web of interactions between protein domains of the Protein Family database (PFAM), which are weighted by a probability score. We apply metrics that combine the static layout and the weights of the underlying interactions. We observe that unweighted measures as well as their weighted counterparts largely share the same trends in the underlying domain interaction network. However, we only find weak signals that weights and the static grid of interactions are connected entities. Therefore assuming that a protein interaction is governed by a single domain interaction, we observe strong and significant correlations of the highest scoring domain interaction and the confidence of protein interactions in the underlying interactions of yeast and fly.

Modeling an interaction between proteins if we find a high scoring protein domain interaction we obtain 1, 428 protein interactions among 361 proteins in the human malaria parasite *Plasmodium falciparum*. Assessing their quality by a logistic regression method we observe that increasing confidence of predicted interactions is accompanied by high scoring domain interactions and elevated levels of functional similarity and evolutionary conservation.

**Conclusion:**

Our results indicate that probability scores are randomly distributed, allowing to treat static grid and weights of domain interactions as separate entities. In particular, these finding confirms earlier observations that a protein interaction is a matter of a single interaction event on domain level. As an immediate application, we show a simple way to predict potential protein interactions by utilizing expectation scores of single domain interactions.

## Background

The depiction of interactions between genes, proteins and metabolites as networks has uncovered unexpected similarities in the organization of various biological networks, indicating that generic principles and mechanics give rise to their structure. Although such networks vary extensively in their complexity, corroborative evidence points to a series of simple organizing principles that characterize all complex networks. The most dramatic is the scale-free nature of these networks, a remarkable inhomogeneity that highlights a small number of highly connected nodes which secure the networks integrity [1]. The special role such proteins play for the stability of protein interaction networks is further indicated by their significant propensity to be simultaneously essential as well as evolutionary conserved [2]. Reflecting their inherent cohesive nature, complex networks are characterized by the accumulation of discernible modules. Such clusters of densely interconnected nodes combine in an overlapping manner, share well defined functions and hubs as the modules connectors [1,3,4]. Similarly to hubs, cohesively bound motifs of protein networks are frequently conserved as a whole, suggesting their role as evolutionary relevant units [5]. While these findings allowed spectacular insights into the inner workings of a cell, biological networks are generally not only determined by their layout of links. In fact, we expect that the heterogeneity in the utilization of connections has a major impact on the organization of cellular activities as well. Recently, attention turned to weighted scientific collaborations and airways networks [6], allowing a first insight into the intricate interplay between links and their weights. Concluding, analysis of real world networks indicate that the static grid of links and their weights can not be regarded as separate entities. Here, we present a first statistical analysis of a weighted biological network by considering a web of PFAM domain interactions. Each link between domains is weighted by an expectation score, reflecting the probability that a particular domain interaction indeed gives rise to observed protein interactions. Applying metrics that combine the static layout of interactions and their weights, we observe that the patterns of correlations are similar for weighted and unweighted network parameters. In contrast to other real world networks, we find weak signals that do not support an entanglement of static grid and weights of domain interactions, allowing us to confirm that a protein interactions are largely governed by single domain interactions.

Assuming that pairs of interacting proteins in *S. cerevisiae *and *D. melanogaster *are indeed dominated by the highest scoring domain interaction their domain architectures suggest, we find that the confidence score of a protein interaction correlates well with its highest scoring domain interaction. As an application, this observation indicates a simple method to model interactions between proteins of the human malaria parasite *P. falciparum*. Assuming an interaction between proteins if we find at least one high scoring domain interaction we predict 1, 428 novel protein interactions among 321 proteins. The quality of each predicted interaction is assessed by a logistic regression model, allowing us to uncover reliable interactions between proteins that share similar functions and are preferably conserved in evolution.

## Results

As a source of high quality interaction data of protein domains we utilized the results of a recent study by Riley et al. [7]. In this statistical approach, called domain pair exclusion analysis (DPEA), a likelihood ratio test is applied to assess the contribution of each potential PFAM-A and PFAM-B domain [8] interaction to the likelihood of a set of observed protein interactions as of DIP [9]. Applying a statistical framework which evaluates the confidence that domains *i *and *j *indeed interact, the authors obtain a network of 1, 566 domains that are embedded in a web of 2, 767 interactions. Weighting each interaction by its probability score – the expectation value [7] – we are primarily interested in the interplay between topology and the reliability of the underlying interactions.

**Figure 1:**
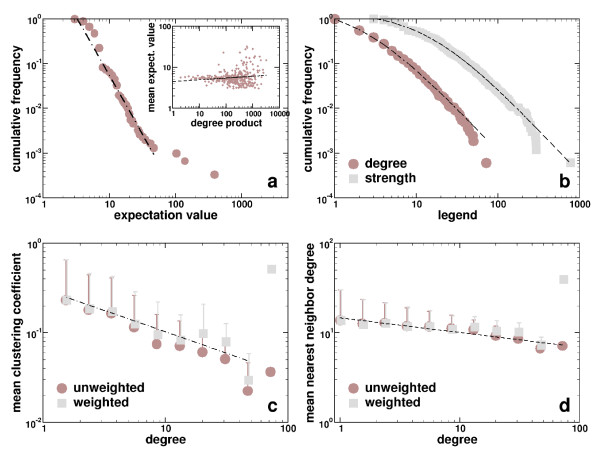
**Statistics of the domain interaction network**. **(a)**. In the cumulative distribution of the expectation value of domain interactions we observe a heavy tail. Focusing on lower ranges of the expectation value, we approximated a power-law (*P*(*E*) ~ *E*^-2.7^). The dependence of an interactions expectation value from the product of the domains degree *k*_*i*_*k*_*j *_shows a weak correlation (inset) (⟨*E*⟩ ~ *k*_*i*_kj−0.04
 MathType@MTEF@5@5@+=feaafiart1ev1aaatCvAUfKttLearuWrP9MDH5MBPbIqV92AaeXatLxBI9gBaebbnrfifHhDYfgasaacH8akY=wiFfYdH8Gipec8Eeeu0xXdbba9frFj0=OqFfea0dXdd9vqai=hGuQ8kuc9pgc9s8qqaq=dirpe0xb9q8qiLsFr0=vr0=vr0dc8meaabaqaciaacaGaaeqabaqabeGadaaakeaacqWGRbWAdaqhaaWcbaGaemOAaOgabaGaeyOeI0IaeGimaaJaeiOla4IaeGimaaJaeGinaqdaaaaa@3438@, Pearson's *r *= 0.30, *P *< 10^-5^, Spearman's rank *ρ *= -0. 17, *P *< 10^-5^, inset). **(b) **For single domain based measures such as the degree *k *and the strength *s*, we observe power-law tailed cumulative frequency distributions as well. Both distributions follow a generalized Zipf's law (*P*(*k*) = 8.7 × (2.1 + *k*)^-1.9^, *P*(*s*) = 298.0 × (14.6 + *s*)^-2.0^). **(c) **Indicating a networks modularity, the dependence of the clustering coeffcient *C *decays as a power-law, *C*(*k*) ~ *k*^-0.55^. Basically, we observe the same correlation for the weighted clustering coeffcient *C*^*w*^(*k*) ~ *k*^-0.47^, indicating that the weighted generalization of the clustering coeffcient does not change the initial correlations. **(d) **The unweighted average nearest neighbor degree slightly decays with increasing degree. This albeit weak dependency is roughly approximated by a power-law (*k*_*nn *_~ *k*^-0.16^). In principle, we obtain the same result for the weighted representation as well (knnw
 MathType@MTEF@5@5@+=feaafiart1ev1aaatCvAUfKttLearuWrP9MDH5MBPbIqV92AaeXatLxBI9gBaebbnrfifHhDYfgasaacH8akY=wiFfYdH8Gipec8Eeeu0xXdbba9frFj0=OqFfea0dXdd9vqai=hGuQ8kuc9pgc9s8qqaq=dirpe0xb9q8qiLsFr0=vr0=vr0dc8meaabaqaciaacaGaaeqabaqabeGadaaakeaacqWGRbWAdaqhaaWcbaGaemOBa4MaemOBa4gabaGaem4DaChaaaaa@3279@ ~ *k*^-0.11^). In (c) and (d), we logarithmically binned the data points and calculated mean values and standard deviations in each bin.

Allowing a first insight in the weights role, we observe a heavy tail in the cumulative distribution of the expectation value of domain links *E*, which can be roughly approximated by a power-law (*P*(*E*) *~ E*^-2.7^) (Figure 1a). In real world networks the correlation of the degree product *k*_*i*_*k*_*j *_and the weight *w*_*ij *_follows a power-law shaped curve, potentially indicating an intricate relationship between the static layout and weights of links. In our case, we hardly find such a dependence (Figure 1a, inset). In fact, the mean expectation value is almost constant for more than two decades, indicating a general lack of correlation between weights and the domains number of interaction partners [6]

Investigating further if the topology of the underlying domain interaction network and their weights are indeed independent from each other, we combine both topology and weights by a series of measures that enable a more significant assessment of the impact of weights [6]. In an unweighted domain interaction network, the domains degree is defined as *k*_*i *_= ∑_*j*_*a*_*ij *_where *a*_*ij *_= 1 if there exists a link between domains *i *and *j*. Extending this definition, the strength of a domain *i *is defined as

si=∑jaijEij,     (1)
 MathType@MTEF@5@5@+=feaafiart1ev1aaatCvAUfKttLearuWrP9MDH5MBPbIqV92AaeXatLxBI9gBaebbnrfifHhDYfgasaacH8akY=wiFfYdH8Gipec8Eeeu0xXdbba9frFj0=OqFfea0dXdd9vqai=hGuQ8kuc9pgc9s8qqaq=dirpe0xb9q8qiLsFr0=vr0=vr0dc8meaabaqaciaacaGaaeqabaqabeGadaaakeaacqWGZbWCdaWgaaWcbaGaemyAaKgabeaakiabg2da9maaqafabaGaemyyae2aaSbaaSqaaiabdMgaPjabdQgaQbqabaGccqWGfbqrdaWgaaWcbaGaemyAaKMaemOAaOgabeaakiabcYcaSaWcbaGaemOAaOgabeqdcqGHris5aOGaaCzcaiaaxMaadaqadaqaaiabigdaXaGaayjkaiaawMcaaaaa@4113@

accounting for individual expectation values *E*_*ij *_as weights of interactions of domain *i*. Comparing the statistical properties of a domains degree *k *and its strength *s *we observe that their frequency distributions follow a generalized Zipf's law *P*(*x*) = *α *× (*β *+ *x*)^-*γ*^(Figure 1b) [10]. The power-law tail of the degree distribution indicates the presence of scale-free topology [11], suggesting that the integrity of the underlying network basically depends on a small subset of highly connected nodes. Analogously, there exists a majority of nodes having low strength while a minority of nodes reach high levels of strength. A list of highest interacting domains shows prominent protagonists that are responsible for important cellular functions such as signaling and cell-cell contacts (Table 1). In particular, we observe that highly connected domains such as pkinase, rrm1 or Zinc finger C2H2 also pool a lot of strength, indicating a proportionality between high level of interactions and their strength.

**Table 1:** Statistics of single domains. Domains in the underlying interaction network are characterized according to their degree *k *and their strength *s*, defined as the sum of all weights the domain in question is involved in. Here, we show the 10 most connected and strongest PFAM domains.

PFAM domain	description	degree *k*	PFAM domain	description	strength *s*
PF01423	LSM	72	PF01423	LSM	777.7
PF00071	ras	50	PF00118	TCP-1/cpn60	294.5
PF00022	actin	50	PF00022	actin	291.5
PF00069	pkinase	49	PF00069	pkinase	289.0
PF00076	rrm1	45	PF00071	ras	263.5
PF00118	TCP-1/cpn60	43	PF00076	rrm1	253.4
PF00096	zf-C2H2	39	PB075870	–	248.8
PB075780	–	39	PF00227	proteasome	237.1
PF00036	efhand	36	PF01008	IF-2B	226.5
PF01008	IF-2B	35	PF00001	7tm-1	226.0

Investigating the local cohesiveness of network areas, the unweighted representation of the clustering coeffcient *C*_*i *_measures the degree of cohesiveness around a particular domain *i *[12]. The dependence of the average clustering coeffcient *C *from the domains degree *k *recovers further information about the structure of the underlying network. In most real world networks *C*(*k*) exhibits a highly nontrivial behavior as exemplified by a power-law decay with increasing degree *k*. Averaging over the clustering coeffcients of domains with a certain degree *k*, we find this particular signature, indicating the presence of a nested hierarchy of modules [1] (Figure 1c). Accounting for weights, Barrat *et al*. [6] extended the initial definition of the clustering coeffcient to combine topological information with weights of network links. Considering the expectation value of each domain interactions *E *as the weight of links, we define the weighted clustering coeffcient as

Ciw=1si(ki−1)∑j,hEij+Eih2aijaihajh.     (2)
 MathType@MTEF@5@5@+=feaafiart1ev1aaatCvAUfKttLearuWrP9MDH5MBPbIqV92AaeXatLxBI9gBaebbnrfifHhDYfgasaacH8akY=wiFfYdH8Gipec8Eeeu0xXdbba9frFj0=OqFfea0dXdd9vqai=hGuQ8kuc9pgc9s8qqaq=dirpe0xb9q8qiLsFr0=vr0=vr0dc8meaabaqaciaacaGaaeqabaqabeGadaaakeaacqWGdbWqdaqhaaWcbaGaemyAaKgabaGaem4DaChaaOGaeyypa0ZaaSaaaeaacqaIXaqmaeaacqWGZbWCdaWgaaWcbaGaemyAaKgabeaakiabcIcaOiabdUgaRnaaBaaaleaacqWGPbqAaeqaaOGaeyOeI0IaeGymaeJaeiykaKcaamaaqafabaWaaSaaaeaacqWGfbqrdaWgaaWcbaGaemyAaKMaemOAaOgabeaakiabgUcaRiabdweafnaaBaaaleaacqWGPbqAcqWGObaAaeqaaaGcbaGaeGOmaidaaaWcbaGaemOAaOMaeiilaWIaemiAaGgabeqdcqGHris5aOGaemyyae2aaSbaaSqaaiabdMgaPjabdQgaQbqabaGccqWGHbqydaWgaaWcbaGaemyAaKMaemiAaGgabeaakiabdggaHnaaBaaaleaacqWGQbGAcqWGObaAaeqaaOGaeiOla4IaaCzcaiaaxMaadaqadaqaaiabikdaYaGaayjkaiaawMcaaaaa@5D36@

Since the structure essentially follows the concept of the original clustering coeffcient, we expect that Ciw
 MathType@MTEF@5@5@+=feaafiart1ev1aaatCvAUfKttLearuWrP9MDH5MBPbIqV92AaeXatLxBI9gBaebbnrfifHhDYfgasaacH8akY=wiFfYdH8Gipec8Eeeu0xXdbba9frFj0=OqFfea0dXdd9vqai=hGuQ8kuc9pgc9s8qqaq=dirpe0xb9q8qiLsFr0=vr0=vr0dc8meaabaqaciaacaGaaeqabaqabeGadaaakeaacqWGdbWqdaqhaaWcbaGaemyAaKgabaGaem4DaChaaaaa@30BA@ retains its dependence from the degree *k*. Indeed, we find a power-law dependence in both networks (Figure 1c). Considering the mean weighted clustering coeffcient of the whole network as the arithmetic mean over all domains *N*, , we obtain 0.097. Comparing this result to the value of the mean unweighted clustering coeffcient of 0.093, we find that ⟨*C*^*w*^⟩/⟨*C*⟩ ≈ 1.0. Since the weighted clustering coeffcient reflects a domain's neighborhood to be connected to domains of similar strength the latter result indicates that local clustering predominately occurs on the level of comparable strength.

Another measure that allows insights in the relationship of network layout and weights are degree-degree correlations. Similarly to *C*^*w*^, we define the average weighted nearest-neighbors degree as [6]



In an unweighted network the definition of *k*_*nn*,*i *_recovers the average nearest neighbor degree of a node, where knn,i=1ki∑jaijkj
 MathType@MTEF@5@5@+=feaafiart1ev1aaatCvAUfKttLearuWrP9MDH5MBPbIqV92AaeXatLxBI9gBaebbnrfifHhDYfgasaacH8akY=wiFfYdH8Gipec8Eeeu0xXdbba9frFj0=OqFfea0dXdd9vqai=hGuQ8kuc9pgc9s8qqaq=dirpe0xb9q8qiLsFr0=vr0=vr0dc8meaabaqaciaacaGaaeqabaqabeGadaaakeaacqWGRbWAdaWgaaWcbaGaemOBa4MaemOBa4MaeiilaWIaemyAaKgabeaakiabg2da9maalaaabaGaeGymaedabaGaem4AaS2aaSbaaSqaaiabdMgaPbqabaaaaOWaaabeaeaacqWGHbqydaWgaaWcbaGaemyAaKMaemOAaOgabeaakiabdUgaRnaaBaaaleaacqWGQbGAaeqaaaqaaiabdQgaQbqab0GaeyyeIuoaaaa@4292@. In the presence of correlations with connectivity *k*, the behavior of the latter measure *k*_*nn*,*i*_(*k*) identifies two classes of networks. If *k*_*nn*_(*k*) is an increasing function with *k*, vertices with higher degree have an increased probability to be connected with large-degree vertices, a feature that is known as assortative mixing. If *k*_*nn*_(*k*) decreases with *k*, the underlying network is disassortative, indicating that high degree vertices predominantly are connected to sparsely linked ones. Similarly to other biological networks [13], we find a weak albeit significant trend toward disassortativity in both the unweighted and weighted domain interaction networks (Figure 1d). Considering the nearest neighbor degree of the whole network as the arithmetic mean over all nodes *N*, 〈knnw〉=1N∑i=1Nknn,iw
 MathType@MTEF@5@5@+=feaafiart1ev1aaatCvAUfKttLearuWrP9MDH5MBPbIqV92AaeXatLxBI9gBaebbnrfifHhDYfgasaacH8akY=wiFfYdH8Gipec8Eeeu0xXdbba9frFj0=OqFfea0dXdd9vqai=hGuQ8kuc9pgc9s8qqaq=dirpe0xb9q8qiLsFr0=vr0=vr0dc8meaabaqaciaacaGaaeqabaqabeGadaaakeaadaaadaqaaiabdUgaRnaaDaaaleaacqWGUbGBcqWGUbGBaeaacqWG3bWDaaaakiaawMYicaGLQmcacqGH9aqpdaWcaaqaaiabigdaXaqaaiabd6eaobaadaaeWaqaaiabdUgaRnaaDaaaleaacqWGUbGBcqWGUbGBcqGGSaalcqWGPbqAaeaacqWG3bWDaaaabaGaemyAaKMaeyypa0JaeGymaedabaGaemOta4eaniabggHiLdaaaa@45F3@, we obtain 12.81. Comparing this result to the value of the mean unweighted nearest neighbor degree of 12.84, we find that 10216;knnw
 MathType@MTEF@5@5@+=feaafiart1ev1aaatCvAUfKttLearuWrP9MDH5MBPbIqV92AaeXatLxBI9gBaebbnrfifHhDYfgasaacH8akY=wiFfYdH8Gipec8Eeeu0xXdbba9frFj0=OqFfea0dXdd9vqai=hGuQ8kuc9pgc9s8qqaq=dirpe0xb9q8qiLsFr0=vr0=vr0dc8meaabaqaciaacaGaaeqabaqabeGadaaakeaacqWGRbWAdaqhaaWcbaGaemOBa4MaemOBa4gabaGaem4DaChaaaaa@3279@10217;/κ_nnˆw≈ 1.0, indeed confirming that in both the weighted as well as unweighted representation the disassortative behavior prevails.

**Figure 2:**
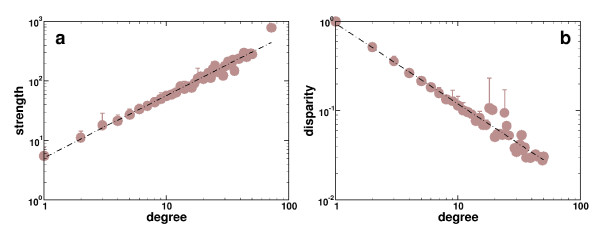
**Statistics of domain strength**. **(a)**. The strength of a domain is defined as the sum of all expectation values of interactions a domain is involved in. The dependence of the strength from the domains degree shows a clear power-law *s*(*k*) ~ *k*^1.04 ^(Pearson's *r *= 0.94, *P *< 10^-5^; Spearman's *ρ *= 0.93, *P *< 10^-5^), suggesting that connectivity and weights are widely independent. **(b) **The disparity value offers further support for this conclusion, since we find that *Y*_2_(*k*) ~ *k*^-0.9 ^(Pearson's *r *= 0.43, *P *< 10^-5^; Spearman's *ρ *= 0.88, *P *< 10^-5^).

The previously introduced topological measures of both unweighted and weighted representations of the same domain interaction network share the same qualitative features, suggesting that weights and topology are entangled entities. However, recalling the observation that the degree product does not correlate with the links underlying weights casts doubt on this assumption. Further insights into a potential interplay of topology and utilization of domain interactions arise from correlations between a domains degree and strength (Figure 2a). Despite the existence of inevitable fluctuations, the dependence of the strength from the degree of a domain in the underlying domain interaction network shows a clear and significant power-law *s*(*k*) *~ k*^*β *^with *^β^*= 1.04, allowing us to conclude that topology and utilization of links in domain interaction networks are separate entities since independent weights and connectivities would lead to an exponent *^β^*= 1 [6]. We receive further support of this hypothesis by the disparity value *Y*_2_, a measure that quantifies biased distributions, defined as

Y2(i)=∑j∈Γ(i)Eij2si2     (4)
 MathType@MTEF@5@5@+=feaafiart1ev1aaatCvAUfKttLearuWrP9MDH5MBPbIqV92AaeXatLxBI9gBaebbnrfifHhDYfgasaacH8akY=wiFfYdH8Gipec8Eeeu0xXdbba9frFj0=OqFfea0dXdd9vqai=hGuQ8kuc9pgc9s8qqaq=dirpe0xb9q8qiLsFr0=vr0=vr0dc8meaabaqaciaacaGaaeqabaqabeGadaaakeaacqWGzbqwdaWgaaWcbaGaeGOmaidabeaakiabcIcaOiabdMgaPjabcMcaPiabg2da9maaqafabaWaaSaaaeaacqWGfbqrdaqhaaWcbaGaemyAaKMaemOAaOgabaGaeGOmaidaaaGcbaGaem4Cam3aa0baaSqaaiabdMgaPbqaaiabikdaYaaaaaaabaGaemOAaOMaeyicI4Saeu4KdCKaeiikaGIaemyAaKMaeiykaKcabeqdcqGHris5aOGaaCzcaiaaxMaadaqadaqaaiabisda0aGaayjkaiaawMcaaaaa@494A@

where Γ(*i*) is the set of neighbors of domain *i*. In Figure 2b we observe a clear power-law in the dependence of the disparity value *Y*_2 _from the degree *k*, *Y*_2_(*k*) ~ *k*^-0.9^. Similarly to the dependence of the strength from the degree (Figure 2a), an exponent close to 1 suggests that the expectation values of domain interactions are distributed in an uncorrelated manner [6,14].

**Figure 3:**
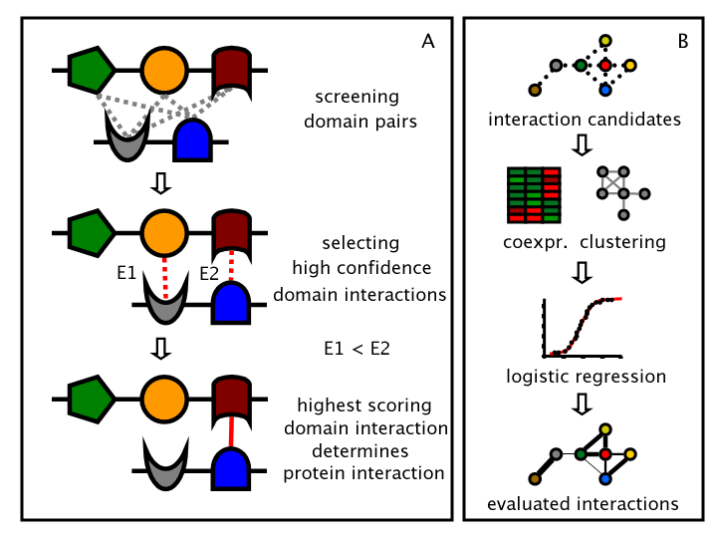
**Schematic illustrations of prediction and evaluation procedures**. **(a)**. Each pair of proteins in *P. falciparum *that shares at least on GO Slim term of the cellular component annotation set is screened for all possible domain pairs. Comparing all interacting domain pairs according to their expectation value, we assume that the highest scoring domain interaction is governing the candidate interaction. **(b) **For each interaction candidate we calculate hypergeometric clustering coeffcients *C*_*vw *_and co-expression correlation coeffcients *r*_*P*_. These parameters allow the domain independent assessment of a protein interaction by utilizing a logistic regression model.

**Figure 4:**
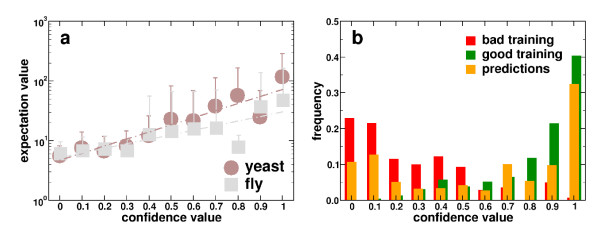
**Expectation score vs. confidence in protein interactions of yeast and fly and statistics of training sets and predictions in Plasmodium**. **(a)**. Assigning each protein interaction the domain interaction with highest expectation value, we observe that the confidence in the underlying protein interaction correlates with the expectation value of the highest scoring interacting domain pair. In particular, the dependence of the mean domain expectation value *E *in each bin of confidence values of yeast protein interactions follows a statistically significant exponential distribution (*E *~ *e*^(2.73 × *cv*)^, Pearson's *r *= 0.28, *P *< 10^-5^, Spearman's *ρ *= 0.31, *P *< 10^-5^). In principle, we obtain similar results for fly protein interactions (inset) (*E *~ *e*^(1.75 × *cv*)^, *r *= 0.19, *P *< 10^-5^; *ρ *= 0.16, *P *< 10^-5^), allowing us to conclude that modeling a protein interaction by the highest scoring domain interaction is a suffcient approximation for the determination of the presence and quality of the underlying protein interaction. Error bars correspond to standard deviations in each bin. **(b) **In order to evaluate predicted interactions in *P. falciparum*, we utilized a logistic regression model that we trained by carefully selected sets of true positive and negative interactions. Binning confidence values, we show the frequencies of the predicted protein interactions, the positive (good) and negative (bad) training sets.

The absence of any correlations between the structure of the web of domain interactions and their confidence suggests that domain interactions hardly interfere with each other. As a consequence, protein interactions are primarily governed by a single domain interaction. Indeed, a recent survey of protein interactions uncovered a rate of 94% that protein interactions are determined by a single pairwise domain interaction [15] while protein interactions that involve interactions between two or more domains are hardly found. A high *E *reflects the probability that the domains in question indeed interact while a low *E*_*ij *_suggests that other potential domain interactions are roughly as good at explaining the observed protein interactions [7]. Therefore, we assume that a protein interaction is governed by the domain interaction with the highest expectation value. In order to uncover a potential correlation between the quality of a particular protein interaction and the highest scoring domain interaction, we utilize two well curated sets of protein interactions in *S. cerevisiae *[16] and *D. melanogaster *[17] where each interaction is evaluated by a confidence score. Utilizing information about the domain composition of proteins as of the Integr8 database, we screen each domain pair that is suggested by the domain architectures of the underlying proteins. Provided these pairs indeed map to high scoring domain interactions, each protein interaction is assumed to be governed by the domain interaction with highest expectation score. Applied to the evaluated protein interaction sets of *S. cerevisae *and *D. melanogaster*, we observe a strong and significant correlation between an interactions confidence and the expectation value of the underlying highest scoring domain interaction (Figure 4a). In turn, we can potentially use the previous conclusion that the absence of correlations between interactions and their probability indicates the dominance of single domain interactions as a means to infer protein interactions. As an organism, we chose the human malaria parasite *P. falciparum*. Utilizing domain information from the Integr8 database we annotate Plasmodium proteins with their corresponding PFAM domains. In order to avoid interactions between proteins that appear in different compartments we additionally assign each protein with its cellular component terms as of the GO Slim database [18]. Considering all protein pairs of Plasmodium we select those that share at least one GO Slim term. The domain architectures of candidate protein pairs are screened for domain pairs that have at least one high scoring domain interaction. In case we find more than one high scoring domain interaction, we choose the highest scoring one, according to the statistical argument that domain interactions with higher expectation score have a better chance to explain the underlying protein interaction. In Figure 3a, we give a schematic survey of the procedure. Applying this method to the proteome of *P. falciparum *we find 1, 428 interactions between 361 proteins [see Additional file 1]. In order to evaluate each of these potential protein interactions, we characterize each link by measures that reflect biological significance. In particular, we are interested in parameters that are independent of the initial assumption that the highest scoring domain interaction indeed can be used to predict protein interactions. As such, we choose co-expression correlation values of interacting proteins, since similar expression profiles tend to indicate interacting proteins. For *P. falciparum*, we utilized gene expression data over 48 time points. Compiling gene expression data derived from micro-array analysis [19-21], we determine Pearson's correlation coeffcients *r*_*P *_of each interaction (see Materials and Methods). In addition, we calculated hypergeometric clustering coeffcients *C*_*vw *_for each interaction, a topological measure that reflects local cohesiveness around a certain link and strongly correlates with the quality of the underlying protein interaction [22] (see Material and Methods). Combining these measures, we utilized a logistic regression method (see Material and Methods) trained by carefully selected sets of 213 true positive and 173 negative interactions, allowing us to assess the quality of each interaction by a confidence score between 0 and 1 (Figure 3b). As a quality measure of the utilized training sets, we performed a leave-one-out strategy, allowing us to obtain 95% accuracy.

**Figure 5:**
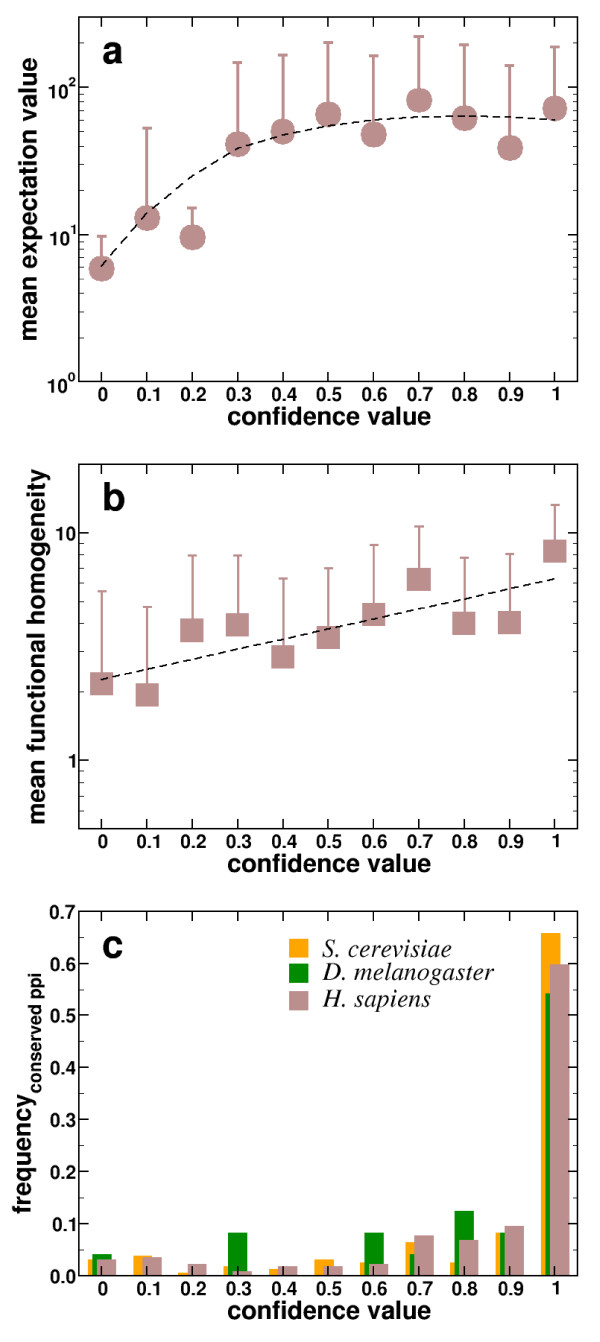
**Statistics of predicted interactions in Plasmodium (a)**. In each bin, we calculated the mean expectation value of domain interactions that govern the underlying protein interactions. In particular we obtain a significant correlation (*r *= 0.22, *P *< 10^-5^, *ρ *= 0.34, *P *< 10^-5^). Error bars correspond to standard deviations in each bin. **(b) **In the same way, we calculated the mean functional homogeneity, a measure that reflects the probability that GO terms of interacting proteins are similar. In particular, we find a statistically significant correlation (*r *= 0.45, *P *< 10^-5^, *ρ *= 0.52, *P *< 10^-5^). **(c) **Determining the frequency of interactions which are fully conserved in the organisms *S. cerevisiae*, *D. melanogaster *and *H. sapiens *we find a strong tendency toward evolutionary conservation of predicted interactions with elevated level of confidence.

Binning interactions according to their confidence value, we observe that about half of the interactions have an elevated degree of confidence (Figure 4b). In each bin, we averaged the expectation score of the domain interactions and observe that high quality of protein interactions – as exemplified by high confidence – are strongly linked to high expectation scores of the underlying domain interaction (Figure 5a). Supported by significant correlation values, this observation is a confirmation of our original assumption that protein interactions are dominated by the highest scoring domain interactions, while high scoring domain interactions indicate the presence of a potential protein interaction. As additional measures of quality, we make use of the well known fact that protein interactions occur between proteins of similar function [23]. As a measure of functional homogeneity of interacting proteins, we apply a hypergeometric test (see Materials and Methods) of the distributions of the proteins GO terms [18]. In particular, this statistical measure reflects the probability that GO terms of interacting proteins have been distributed randomly. Averaging over all interaction specific values in each bin, we find a strong and significant correlation, confirming that protein interactions of increasing confidence tend to occur between functionally related proteins (Figure 5b). As a final test, we wondered if the predicted protein interactions in *P. falciparum *have an evolutionary signature. In particular, we utilized three protein interaction sets of the organisms *S. cerevisiae *[16], *D. melanogaster *[17] and *H. sapiens *[23,24]. Utilizing orthologous protein information from the InParanoid database [25], we sampled all protein interactions in each organisms that have a fully conserved counterpart – an interolog [26] – in the predicted set of interactions of *P. falciparum*. In Figure 5c, we observe that especially predictions with high confidence pool most of the found interologs in each organism, strongly indicating the reliability of our predictions.

We compared the predicted sets of interactions to a recently published set of experimentally determined protein interactions of *P. falciparum *[27]. Although many interactions of this set have been assigned potential protein domain interactions, the utilized domain information does not overlap strongly with PFAM, restricting the overlap with our predicted set to only 2 interactions. In particular, we find self interactions of the hypothetical Plasmodium proteins PFL0275w and PF10_0232. In the first case, a self interaction of the FHA domain gives rise to the observed protein interaction, while a self interaction of chromo domain determines the latter one. In both cases, the interacting proteins are hypothetical, meaning that their function is unclear. However, the fact that we found domain interactions suggests a role for these proteins. In particular, the forkhead-associated FHA domain is a phosphopeptide recognition domain found in many regulatory proteins, while the chromo (CHRromatin Organization MOdifier) domain is a conserved region of around 60 amino acids involved in the alteration of the structure of chromatin. Putatively, PFL0275w is involved in regulatory activities while PF10_0232 might play a role in chromatin remodeling. In general, our predictions show a prevalence of functions revolving around the proteasome, spliceosome and ribosome. In particular, Table 2 ranks the domain interactions that gave rise to the highest number of predictions in *P. falciparum*. In particular, we observe that domain interactions between the RNA recognition motif rrm1, proteasome and LSM domains appear among the most prevalent domain interactions. As the previous examples illustrates, many interactions are related to self interactions of the underlying domains. As such, we observe a total of 154 self interactions. Indeed, it is well known that multi-protein complexes contain homo-dimers including proteasome [28], ribosome [29] and spliceosome [30]. In particular, rrm's are found in a variety of RNA binding proteins, including various hnRNP proteins, proteins implicated in regulation of alternative splicing, and protein components of snRNPs. The LSM domain contains Sm proteins as well as other related LSM (Like Sm) proteins. The U1, U2, U4/U6, and U5 small nuclear ribonucleoprotein particles (snRNPs) involved in pre-mRNA splicing contain seven Sm proteins in common, which assemble around the Sm site present in four of the major spliceosomal small nuclear RNAs. The U6 snRNP binds to the LSM (Like Sm) proteins. The proteasome is a multicatalytic proteinase complex that is involved in an ATP/ubiquitin-dependent proteolytic pathway. In eukaryotes, the proteasome is composed of about 28 distinct subunits, which form a highly ordered ring-shaped structure (20S ring). Concluding, in the proteasome, ribosome and spliceosome proteins which carry those domains tend to shape stable structures which are mostly governed by self domain interactions, validating the presence of self interactions in our predictions.

**Table 2:** Domain interactions in predictions of protein interactions in Plasmodium. Predicting protein interactions by their highest scoring domain interaction in *P. falciparum *we find the following 20 most frequent domain interactions. *N *refers to the domain interactions occurrence in the predicted set, %_*sl *_depicts the percentage of self protein interactions, and *E *is the expectation value of the underlying domain interaction.

domain	description	domain	description	*N*	%_*sl*_	*E*
PF00076	rrm1	PF01423	LSM	137	-	14.5
PF00227	proteasome	PF00227	proteasome	120	12.5	103.1
PF01423	LSM	PF01423	LSM	120	12.5	387.1
PF00005	ABC transporter	PF00005	ABC transporter	83	16.5	4.9
PF00097	zf-C3HC4	PF00240	ubiquitin	74	-	5.7
PF00076	rrm1	PF00076	rrm1	56	28.7	14.5
PF00022	actin	PF00022	actin	55	18.1	8.5
PF00125	histone	PF00125	histone	36	22.1	11.6
PF01423	LSM	PF06220	zf-U1	30	-	20.3
PF02953	Tim10/DDP zinc finger	PF00153	mitochondrial carrier	30	-	6.6
PF00097	zf-C3HC4	PF01283	Ribosomal protein S	24	-	3.0
PF00097	zf-C3HC4	PF01775	Ribosomal L18ae	24	-	7.3
PF00097	zf-C3HC4	PF00833	Ribosomal S17	24	-	3.6
PF00097	zf-C3HC4	PF00827	Ribosomal L15	24	-	3.2
PF00118	TCP-1/cpn60 chaperonin	PF00118	TCP-1/cpn60 chaperonin	23	34.8	17.9
PF00928	Adaptor complexes	PF01217	Clathrin adaptor	20	-	21.7
PF00076	rrm1	PF01974	tRNA intron endonuclease	18	-	3.0
PF00076	rrm1	PF06220	zf-U1	18	-	6.2
PF01602	Adaptin N terminal region	PF01217	Clathrin adaptor complex	16	-	9.2
PF00125	histone	PF00956	Nucleosome assembly protein	16	-	14.5

## Discussion & conclusion

Assessing the statistical characteristics of a weighted domain interaction network we show that the confidence as exemplified by the expectation value of domain interactions is far from being evenly distributed. Characterizing the underlying weighted domain interactions network, we observe that weighted and unweighted measures of topology follow the same trends. Despite these observations we do not find any significant proof that topology and weights in the domain interaction network are necessarily dependent from each other. In fact, correlations between strength and connectivity as well as disparity suggest that weights as exemplified by the expectation score of each domain interaction are randomly distributed, allowing us to (i) treat the static layout of links and their weights as separate entities and (ii) conclude that protein interactions are indeed governed by a single protein domain interaction [15].

The presence of highly reliable domain interactions offers potential new ways for the prediction and evaluation of protein interactions. In particular, we observe a correlation between an elevated confidence level of a protein interaction in yeast and fly and an increase in the reliability of the underlying domain interactions. As an application, we propose a novel method for the inference of potential protein interactions. While this method can be applied to the prediction of protein interactions in any organism for which PFAM annotation of the organisms proteome is available, we chose the human malaria parasite *P. falciparum*. Screening through all pairs of proteins that provide at least one high scoring domain interaction, we sample potential candidates. Here, we stress that the determination of a high scoring domain interaction has been used as a preselection step of potential protein interaction candidates. In order to evaluate each interaction we resort to interaction specific parameters that are independent from the underlying domain interactions. We find interactions between proteins, that not only show an elevated degree of functional similarity and evolutionary conservation, but also validate our assumption that high scoring domain interactions indeed give rise to reliable interactions. Predominately, we find an enrichment of protein interactions caused by domain interactions that represent functions in the ribosome, proteasome and spliceosome. As reported in protein complexes in other eukaryotes, these functions emphasize a considerable amount of self interactions, we also find in our predictions.

Comparing with existing experimental data sets, we only find a minimal overlap, caused by the fact that many proteins of *P. falciparum *currently are not annotated with PFAM domains. On the other hand, experimental determination of protein interactions in *P. falciparum *is in its starting phase covering about a quarter of known proteins. As such, our predictions can help focus experimental studies on specific interactions unique to this pathogen.

## Methods

### Domain-domain interactions

As a source of high quality interaction data of protein domains we utilized the results of a recent study by Riley et al. [7]. In this statistical approach called domain pair exclusion analysis (DPEA), a likelihood ratio test is applied to assess the contribution of each potential PFAM-A and PFAM-B domain [8] interaction to the likelihood of a set of observed protein interactions. DPEA consists of three steps: (i) Utilizing protein interaction data from DIP [9], the frequency *S*_*ij *_of an interaction between *i *and *j *in relation to their abundance in the data is computed. (ii) Using *S*_*ij *_as an initial guess, an expectation maximization algorithm is applied to obtain a maximum likelihood estimate of Θ_*ij *_which stands for the probability of domain interaction *ij *among all the possible domain interactions which are suggested by the domain architectures of the interacting protein pairs where domain *i *and *j *co-occur. In a third step, all possible interactions of domains *i *and *j *are excluded from the mixture of competing hypotheses for the presence of corresponding protein interactions, EM is rerun, and the change in likelihood is expressed as a log odds score, *E*_*ij*_, reflecting the confidence that domains *i *and *j *indeed interact. As such, a high value of *E*_*ij *_indicates that there is extensive evidence in protein interaction data that domains *i *and *j *interact while a low *E*_*ij *_suggests that other potential domain interactions are roughly as good at explaining the observed protein interactions [7]. As a proof of concept, domain pairs inferred to interact with high *E *are significantly enriched among domain pairs known to interact in the Protein Data Bank (PDB). The domain interaction network thus obtained comprises 1, 566 domains which are embedded in 2, 767 interactions that score *E*_*ij *_≥ 3.

### Protein interactions

We utilized a large scale compilation of yeast protein interactions. In particular, this data set combines 47, 783 experimentally obtained protein interactions among 4, 175 proteins in *S. cerevisiae *[16] obtained from sources as diverse as mRNA expression studies and yeast2hybrid screens. Each interaction was characterized by a confidence score obtained by the application of a logistic regression model. Analogously, the quality of experimentally protein interactions in *D. melanogaster *was assessed, allowing for 6, 222 proteins and 16, 914 links [17]. As for direct experimental observations of protein interactions in *P. falciparum*, we utilized a set of 2, 475 interactions among 1, 304 proteins that have been obtained by the modification of a yeast2hybrid method [27]. Additionally, we utilized a large-scale compilation of human interactions totaling 89, 572 interactions among 9, 018 proteins [23,24].

### Protein domain data

The advent of fully sequenced genomes of various organisms has facilitated the investigation of proteomes. The Integr8 database has been set up to provide comprehensive statistical and comparative analyzes of complete proteomes of fully sequenced organisms. The initial version of the application contained data for genomes and proteomes of 182 sequenced organisms (including 19 archae, 150 bacteria and 13 eukaryotes) and proteome analyzes derived through the integration of UniProt [31], InterPro [32], CluSTr [33], GO/GOA [34], EMSD, Genome Reviews and IPI [35]. In particular, we utilized IPI (International Protein Index) files to elucidate the domain architecture of the corresponding proteins. For our analysis, we focused on domain data retrieved from the PFAM database, a reliable collection of multiple sequence alignments of protein families and profile hidden Markov models [36].

### Orthologous protein data

The InParanoid database [25] provides putative orthologous sequence information for the complete proteomes of organism pairs *S. cerevisiae*, *D. melanogaster*, *H. sapiens *and *P. falciparum*. The algorithm for detecting orthologous relationships is based on pairwise similarity scores which are by default calculated with the BLASTP program. InParanoid detects mutual best hits between sequences from two different species. These are two main orthologs that form an orthologous group. Other sequences are added to this group if they are closely related to one of the main orthologs. These members of the orthologous group are called in-paralogs. A confidence value provided by a standard bootstrap procedure for each in-paralog shows how closely related it is to the main ortholog. In our study, we only selected the main sequence pairs of each orthologous group allowing us to obtain 2, 319 yeast proteins, 1, 351 in *D. melanogaster *and 1, 525 in *H. sapiens *with putative orthologs in *P. falciparum*.

### Co-expression data

Genes with similar expression profiles are likely encoding interacting proteins. For *P. falciparum*, we utilized gene expression data, compiling 5, 156 genes over 48 time points as of Winzeler et al., [19,21] and of Bozdech et al. collecting 4, 318 genes over 48 time points [37]. As a gene similarity metric we calculated Pearson's correlation coeffcient for every protein interaction over *m *time points defined as

rp=1m∑i=1mxiyi−〈x〉〈y〉σiσj     (5)
 MathType@MTEF@5@5@+=feaafiart1ev1aaatCvAUfKttLearuWrP9MDH5MBPbIqV92AaeXatLxBI9gBaebbnrfifHhDYfgasaacH8akY=wiFfYdH8Gipec8Eeeu0xXdbba9frFj0=OqFfea0dXdd9vqai=hGuQ8kuc9pgc9s8qqaq=dirpe0xb9q8qiLsFr0=vr0=vr0dc8meaabaqaciaacaGaaeqabaqabeGadaaakeaacqWGYbGCdaWgaaWcbaGaemiCaahabeaakiabg2da9maalaaabaWaaSaaaeaacqaIXaqmaeaacqWGTbqBaaWaaabmaeaacqWG4baEdaWgaaWcbaGaemyAaKgabeaakiabdMha5naaBaaaleaacqWGPbqAaeqaaOGaeyOeI0YaaaWaaeaacqWG4baEaiaawMYicaGLQmcadaaadaqaaiabdMha5bGaayzkJiaawQYiaaWcbaGaemyAaKMaeyypa0JaeGymaedabaGaemyBa0ganiabggHiLdaakeaaiiGacqWFdpWCdaWgaaWcbaGaemyAaKgabeaakiab=n8aZnaaBaaaleaacqWGQbGAaeqaaaaakiaaxMaacaWLjaWaaeWaaeaacqaI1aqnaiaawIcacaGLPaaaaaa@51F9@

where ⟨*x*⟩ and ⟨*y*⟩ are the sample means of expression values *x*_*i *_and *x*_*j*_, and *σ*_*i *_and *σ*_*j *_are their standard deviations.

### Logistic regression

In order to get an estimate of an interactions reliability, we employed a logistic regression model. According to the logistic regression, the probability of a true interaction *T*_*vw *_given the two input variables, hypergeometric clustering coeffcient *x*_1 _= *C*_*vw *_and co-expression correlation coeffcient *x*_2 _= *r*_*P*_, *X *= (*x*_1_*, x*_2_)

Pr(Tvw|X)=exp(β0+β1x1+β2x2)1+exp(β0+β1x1+β2x2)     (6)
 MathType@MTEF@5@5@+=feaafiart1ev1aaatCvAUfKttLearuWrP9MDH5MBPbIqV92AaeXatLxBI9gBaebbnrfifHhDYfgasaacH8akY=wiFfYdH8Gipec8Eeeu0xXdbba9frFj0=OqFfea0dXdd9vqai=hGuQ8kuc9pgc9s8qqaq=dirpe0xb9q8qiLsFr0=vr0=vr0dc8meaabaqaciaacaGaaeqabaqabeGadaaakeaaieGacqWFqbaucqWFYbGCcqGGOaakcqWGubavdaWgaaWcbaGaemODayNaem4DaChabeaakiabcYha8jabdIfayjabcMcaPiabg2da9maalaaabaGae8xzauMae8hEaGNae8hCaaNaeiikaGccciGae4NSdi2aaSbaaSqaaiabicdaWaqabaGccqGHRaWkcqGFYoGydaWgaaWcbaGaeGymaedabeaakiabdIha4naaBaaaleaacqaIXaqmaeqaaOGaey4kaSIae4NSdi2aaSbaaSqaaiabikdaYaqabaGccqWG4baEdaWgaaWcbaGaeGOmaidabeaakiabcMcaPaqaaiabigdaXiabgUcaRiab=vgaLjab=Hha4jab=bhaWjabcIcaOiab+j7aInaaBaaaleaacqaIWaamaeqaaOGaey4kaSIae4NSdi2aaSbaaSqaaiabigdaXaqabaGccqWG4baEdaWgaaWcbaGaeGymaedabeaakiabgUcaRiab+j7aInaaBaaaleaacqaIYaGmaeqaaOGaemiEaG3aaSbaaSqaaiabikdaYaqabaGccqGGPaqkaaGaaCzcaiaaxMaadaqadaqaaiabiAda2aGaayjkaiaawMcaaaaa@6902@

where *β*_*n *_are the parameters of the distribution. Given training data we optimized the distribution parameters by maximizing the likelihood of the data. Here, we applied the corresponding routines as of the Biopython package [38]. As a training set for true positives we choose 213 high scoring protein-interactions in yeast [16] that are fully conserved in Plasmodium. In the same way, we selected 173 low scoring interactions as true negative training set. Applying a leave-one-out analysis to determine the prediction accuracy, our model is recalculated from the training data after removing the interaction to be predicted (leave-one-out), allowing us to obtain the correct result in 95% of cases.

### Hypergeometric clustering coeffcient

Recently, a network topology based approach uncovered a remarkable correlation between enhanced quality of protein interactions and the degree of clustering of their immediate network neighborhood [22]. Considering a network with *N *nodes, we define the hypergeometric clustering coeffcient as

Cvw=−log⁡∑i=|N(v)∩N(w)|min⁡(|N(v)|,|N(w)|)(|N(v)|i)(N−|N(v)||N(w)|−i)(N|N(w)|)     (7)
 MathType@MTEF@5@5@+=feaafiart1ev1aaatCvAUfKttLearuWrP9MDH5MBPbIqV92AaeXatLxBI9gBaebbnrfifHhDYfgasaacH8akY=wiFfYdH8Gipec8Eeeu0xXdbba9frFj0=OqFfea0dXdd9vqai=hGuQ8kuc9pgc9s8qqaq=dirpe0xb9q8qiLsFr0=vr0=vr0dc8meaabaqaciaacaGaaeqabaqabeGadaaakeaacqWGdbWqdaWgaaWcbaGaemODayNaem4DaChabeaakiabg2da9iabgkHiTiGbcYgaSjabc+gaVjabcEgaNnaaqahabaWaaSaaaeaadaqadaqaauaabeqaceaaaeaacqGG8baFcqWGobGtcqGGOaakcqWG2bGDcqGGPaqkcqGG8baFaeaacqWGPbqAaaaacaGLOaGaayzkaaWaaeWaaeaafaqabeGabaaabaGaemOta4KaeyOeI0IaeiiFaWNaemOta4KaeiikaGIaemODayNaeiykaKIaeiiFaWhabaGaeiiFaWNaemOta4KaeiikaGIaem4DaCNaeiykaKIaeiiFaWNaeyOeI0IaemyAaKgaaaGaayjkaiaawMcaaaqaamaabmaabaqbaeqabiqaaaqaaiabd6eaobqaaiabcYha8jabd6eaojabcIcaOiabdEha3jabcMcaPiabcYha8baaaiaawIcacaGLPaaaaaGaaCzcaiaaxMaadaqadaqaaiabiEda3aGaayjkaiaawMcaaaWcbaGaemyAaKMaeyypa0JaeiiFaWNaemOta4KaeiikaGIaemODayNaeiykaKIaeyykICSaemOta4KaeiikaGIaem4DaCNaeiykaKIaeiiFaWhabaGagiyBa0MaeiyAaKMaeiOBa4MaeiikaGIaeiiFaWNaemOta4KaeiikaGIaemODayNaeiykaKIaeiiFaWNaeiilaWIaeiiFaWNaemOta4KaeiikaGIaem4DaCNaeiykaKIaeiiFaWNaeiykaKcaniabggHiLdaaaa@8AC0@

where *N*(*x*) represents the neighborhood of a vertex *x*. Given fixed neighborhood sizes *N*(*v*) and *N*(*w*) of nodes *v *and *w*, the hypergeometric clustering coeffcient increases with elevated overlap between the nodes neighborhoods. Provided that the neighborhoods are independent, the summation can be interpreted as a *p *value, reflecting the probability of obtaining a number of mutual neighbors between nodes *v *and *w *at or above the observed number by chance.

### GO annotation data and functional homogeneity

Similarly to the hypergeometric clustering coeffcient, we define the functional homogeneity of a domain pair *ij*

fhij=−log⁡∑i=|GO(v)∩GO(w)|min⁡(|GO(v)|,|GO(w)|)(|GO(v)|i)(T−|GO(v)||GO(w)|−i)(T|GO(w)|)     (8)
 MathType@MTEF@5@5@+=feaafiart1ev1aaatCvAUfKttLearuWrP9MDH5MBPbIqV92AaeXatLxBI9gBaebbnrfifHhDYfgasaacH8akY=wiFfYdH8Gipec8Eeeu0xXdbba9frFj0=OqFfea0dXdd9vqai=hGuQ8kuc9pgc9s8qqaq=dirpe0xb9q8qiLsFr0=vr0=vr0dc8meaabaqaciaacaGaaeqabaqabeGadaaakeaacqWGMbGzcqWGObaAdaWgaaWcbaGaemyAaKMaemOAaOgabeaakiabg2da9iabgkHiTiGbcYgaSjabc+gaVjabcEgaNnaaqahabaWaaSaaaeaadaqadaqaauaabeqaceaaaeaacqGG8baFcqWGhbWrcqWGpbWtcqGGOaakcqWG2bGDcqGGPaqkcqGG8baFaeaacqWGPbqAaaaacaGLOaGaayzkaaWaaeWaaeaafaqabeGabaaabaGaemivaqLaeyOeI0IaeiiFaWNaem4raCKaem4ta8KaeiikaGIaemODayNaeiykaKIaeiiFaWhabaGaeiiFaWNaem4raCKaem4ta8KaeiikaGIaem4DaCNaeiykaKIaeiiFaWNaeyOeI0IaemyAaKgaaaGaayjkaiaawMcaaaqaamaabmaabaqbaeqabiqaaaqaaiabdsfaubqaaiabcYha8jabdEeahjabd+eapjabcIcaOiabdEha3jabcMcaPiabcYha8baaaiaawIcacaGLPaaaaaGaaCzcaiaaxMaadaqadaqaaiabiIda4aGaayjkaiaawMcaaaWcbaGaemyAaKMaeyypa0JaeiiFaWNaem4raCKaem4ta8KaeiikaGIaemODayNaeiykaKIaeyykICSaem4raCKaem4ta8KaeiikaGIaem4DaCNaeiykaKIaeiiFaWhabaGagiyBa0MaeiyAaKMaeiOBa4MaeiikaGIaeiiFaWNaem4raCKaem4ta8KaeiikaGIaemODayNaeiykaKIaeiiFaWNaeiilaWIaeiiFaWNaem4raCKaem4ta8KaeiikaGIaem4DaCNaeiykaKIaeiiFaWNaeiykaKcaniabggHiLdaaaa@950D@

where *GO*(*i*) is the set of GO Terms of protein *i*, and *T *is the total number of different GO terms [18]. In analogy, the summation can be interpreted as a *p *value, reflecting the probability that a protein pair shares a certain number of GO terms at or above the observed number by chance.

## Supplementary Material

Additional File 1 **Predicted protein interactions in *P. falciparum***. This file contains the full set of predicted protein interactions in *P. falciparum*. Each column is tab-delineated, presenting the interacting proteins (column 1&2), the underlying domain interactions (columns 3 & 4), their expectation score (column 5), the interactions confidence score (column 6) and the proteins functional annotation (columns 7 & 8) Click here for file
